# Antipsychotics increase steroidogenic enzyme gene expression in the rat brainstem

**DOI:** 10.1007/s11033-021-06943-4

**Published:** 2021-11-19

**Authors:** Katarzyna Bogus, Małgorzata Żarczyńska, Artur Pałasz, Aleksandra Suszka-Świtek, John J. Worthington, Marek Krzystanek, Piotr Żarczyński

**Affiliations:** 1 Department of Histology, Faculty of Medical Sciences in Katowice, Medical University of Silesia, ul. Medyków 18, 40-752 Katowice, Poland; 2 Division of Biomedical and Life Sciences, Faculty of Health and Medicine, Lancaster University, Lancaster, LA1 4YG UK; 3 Clinic of Psychiatric Rehabilitation, Department of Psychiatry and Psychotherapy, Faculty of Medical Sciences in Katowice, Medical University of Silesia, ul. Ziolowa 45/47, 40-635 Katowice, Poland

**Keywords:** Neurosteroids, Aromatase, 3β-HSD, P450scc, Olanzapine, Haloperidol

## Abstract

**Background:**

Neurosteroids are involved in several important brain functions and have recently been considered novel players in the mechanic actions of neuropsychiatric drugs. There are no reports of murine studies focusing on the effect of chronic neurosteroid treatment in parallel with antipsychotics on key steroidogenic enzyme expression and we therefore focused on steroidogenic enzyme gene expression in the brainstem of rats chronically treated with olanzapine and haloperidol.

**Methods and results:**

Studies were carried out on adult, male Sprague–Dawley rats which were divided into 3 groups: control and experimental animals treated with olanzapine or haloperidol. Total mRNA was isolated from homogenized brainstem samples for RealTime-PCR to estimate gene expression of related aromatase, 3β-HSD and P450scc. Long-term treatment with the selected antipsychotics was reflected in the modulation of steroidogenic enzyme gene expression in the examined brainstem region; with both olanzapine and haloperidol increasing aromatase, 3β-HSD and P450scc gene expression.

**Conclusions:**

The present findings shed new light on the pharmacology of antipsychotics and suggest the existence of possible regulatory interplay between neuroleptic action and steroidogenesis at the level of brainstem neuronal centres.

## Introduction

**Fig. 1:**
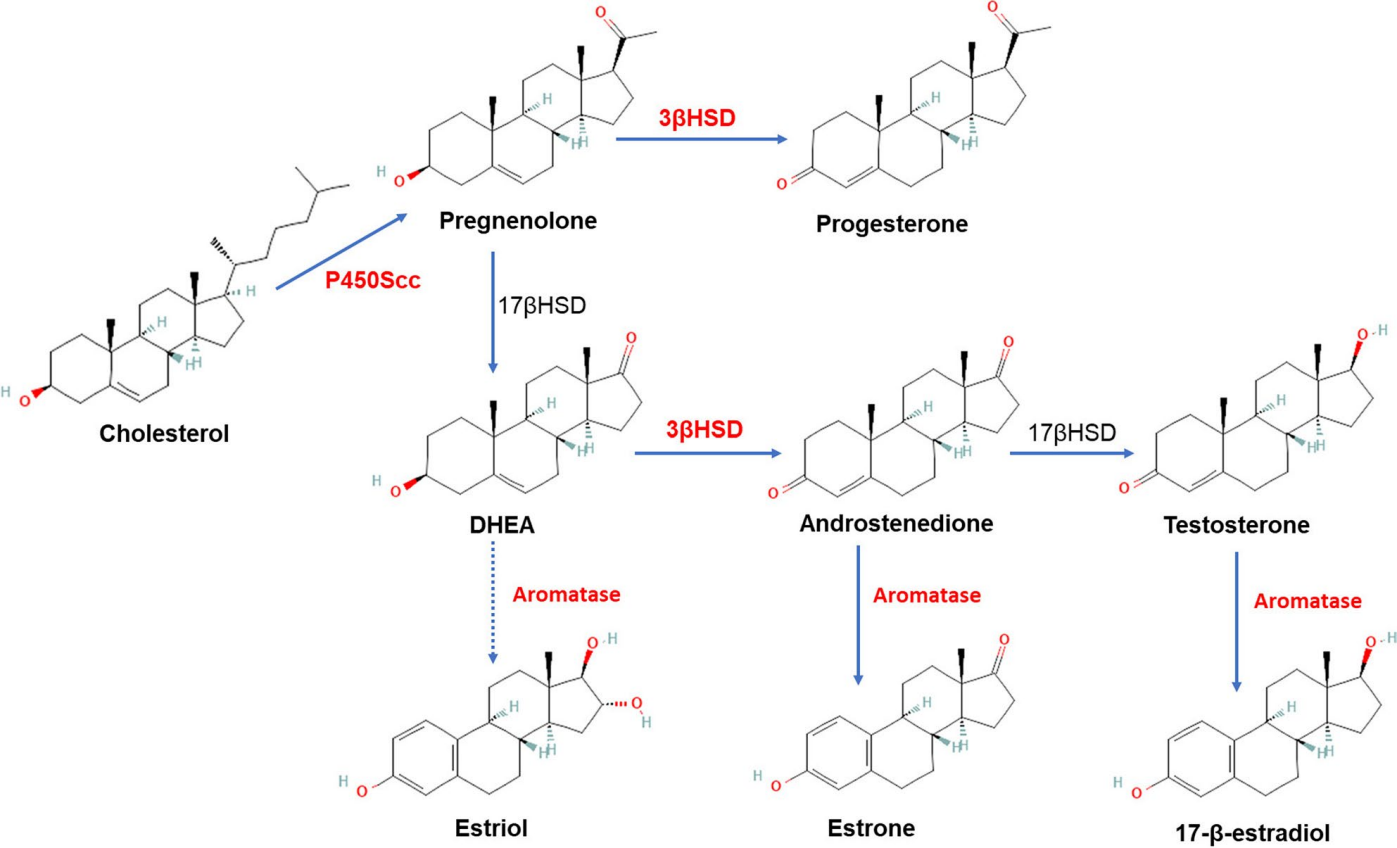
Schematic diagram of the main neurosteroid synthesis pathways. The names of the neurosteroids and metabolites are beneath the structural formulas. Next to each arrow are located names of the enzymes responsible for specific reaction. Dotted one shows the multistage process. Analysed enzymes are in red colour. Enzymes include: cytochrome P450 side-chain cleavage (P450 Scc), Aromatase (CYP19A1), 3β-hydroxysteroid dehydrogenase (3β-HSD), 17β-hydroxysteroid dehydrogenase (17β-HSD). (Color figure online)

Steroids play a crucial role in maintaining and regulating the body’s function, mediating many important physiological processes, such as reproduction, sexual differentiation, ionic and carbohydrate homeostasis and stress responses. In a number of brain structures, these hormones are responsible for numerous neurochemical processes both during development and in adulthood [1]. Regardless of tissue type, all steroid hormones are synthesized from cholesterol as a common precursor molecule. The term "neurosteroids" has been applied to steroids that can be synthesized de novo in the brain and occurs in the central and peripheral nervous systems, occurring both in neurons and glial cells [1]. The first step in the synthesis of steroid hormones is the conversion of cholesterol to pregnenolone (PREG), a process catalyzed by cytochrome P450scc (CYP11A1) (Fig. 1). Mitochondrial P450scc is synthesized in all known steroidogenic tissues, including the brain, with its expression most abundant in the cortex, amygdala, hippocampus and midbrain of the adult rat [2]. Noteworthy, the P450scc protein is detected at very early stages of brain development in the organizing neural tube, prior to adrenal or gonadal organogenesis, which potentially suggests a role for neurosteroids in embryonic neurogenesis. 3β-hydroxysteroid dehydrogenase (3β-HSD) is a membrane bound enzyme located in the endoplasmic reticulum and mitochondria and is responsible for the oxidation and isomerization of inactive ∆5-3β-hydroxysteroids, including pregnenolone and dehydroepiandrosterone (DHEA), to active ∆4-keto steroids—progesterone and androstenedione (AE), respectively (Fig. 1). At least two forms of human 3β-HSD exist, while in rodents four forms of the enzyme have been identified. Rat 3β-HSD is encoded by a wide variety of genes located on chromosome 2, all of which code for protein whose expression is tissue specific. It has been shown that in rats, 3β-HSD mRNA is expressed in several areas of the brain, in particular: the olfactory bulb, striatum, cortex, thalamus, hypothalamus, septum, hippocampus and cerebellum with the transcripts of 3β-HSD mRNA in the rat brain being significantly lower than in the ovary, adrenal glands or liver [3]. In contrast, the human gene for type I 3β-HSD is expressed in the placenta, skin, mammary gland and other organs including the brain, while the human gene encoding type II 3β-HSD is expressed in the adrenal glands and gonads [3]. Aromatase P450 (CYP19A1) is a microsomal enzyme that plays a key role in the biosynthesis of estrogens. Aromatase is a glycoprotein located in the endoplasmic reticulum where it is responsible for the conversion of testosterone to estradiol, androstenedione to estrone, and 16α-hydroxy-dehydroepiandrosterone to estriol (Fig. 1) [4]. Human P450 aromatase is expressed in steroidogenic tissues such as the ovary and placenta, as well as in non-steroidogenic tissues such as adipose tissue and brain [4]. Aromatase is an enzyme necessary for the proper functioning of the body, as evidenced by numerous studies on mutations that inhibit CYP19A1 activity.

Olanzapine is classified as a second-generation antipsychotic drug, referred to as atypical. They are still widely in use due to the smaller number of extrapyramidal side effects and less marked interactions with coadministered drugs. Olanzapine acts as an antagonist of dopamine D_1_–D_5_ receptors in the mesolimbic pathway blocking dopamine signalling on postsynaptic receptors, which has been used in the treatment of positive symptoms in patients suffering from schizophrenia. Olanzapine also exhibits an affinity to serotonin receptors 5HT_2A_, 5-HT_2C_, 5-HT_6_, 5-HT_7_, histamine H_1_, α_1_-adrenergic and muscarinic M_1_–M_5_ receptors. Haloperidol belongs to the first generation (typical) antipsychotic drugs and is still used in other diseases, for example to counter the effects of dopamine and to control psychosis in Huntington's disease. First generation antipsychotics are usually D_2_ receptor antagonists and they may have a slight effect on serotoninergic 5-HT_2_ receptors which improves their pharmacological efficacy, but at the same time causes many side effects. Haloperidol is best known for its antagonism at dopamine D_2_ receptors, with additional antagonism at 5-HT_2A_ receptors, αα1A receptors, α_1B_ receptors and the σ1 receptor [5]. At the level of neural circuits, haloperidol acts as a dopaminergic antagonist in the basal ganglia and it seems that the thalamocortical changes are secondary to the drug's action in this area of the brain [6].

The brainstem is a general term for the unique and relatively small brain structure which plays a fundamental role in maintaining fundamental living functions such as circulation and respiration. A lot of the brainstem’s neural assemblies, including aminergic reticular formation and numerous nuclei of the cranial nerves, are also involved in diverse neurophysiological processes such as learning, memory, feeding regulation, osmotic balance, reproduction, affective reactions, circadian rhythm and pain transmission. Reticular formation consists of several perikarya that produce fundamental brain neurotransmitters such as catecholamines, acetylcholine or serotonin (raphe nuclei). Antipsychotics may hypothetically alter serotonin and/or norepinephrine signaling at the level of aforementioned brainstem neurons. However, at present there is little information about steroidogenesis in the brainstem. An expression of P450sc and 3α-hydroxy, 5α-reduced steroids has been detected in the rat vestibular nuclei and reticular formation suggesting that neurosteroids are involved in the activity of these structures [7]. The effects of DHEA and its derivatives on GABA-ergic regulation of raphe nuclei serotoninergic neurons may underlie some functional properties e.g. behavioural and affective of brainstem neurosteroids [8].

To date, very little is known about the effects of antipsychotic administration on neurosteroidogenic enzyme expression in the brain. The aim of this study was therefore to investigate the effect of long-term treatment with olanzapine and haloperidol on the expression of aromatase, 3-βHSD and P450scc genes in the rat brainstem. This work may shed a new light on antipsychotic pharmacology and help to find possible, neurosteroid-related mechanisms of neuroleptic action in animal models.

## Materials and methods

**Fig. 2:**
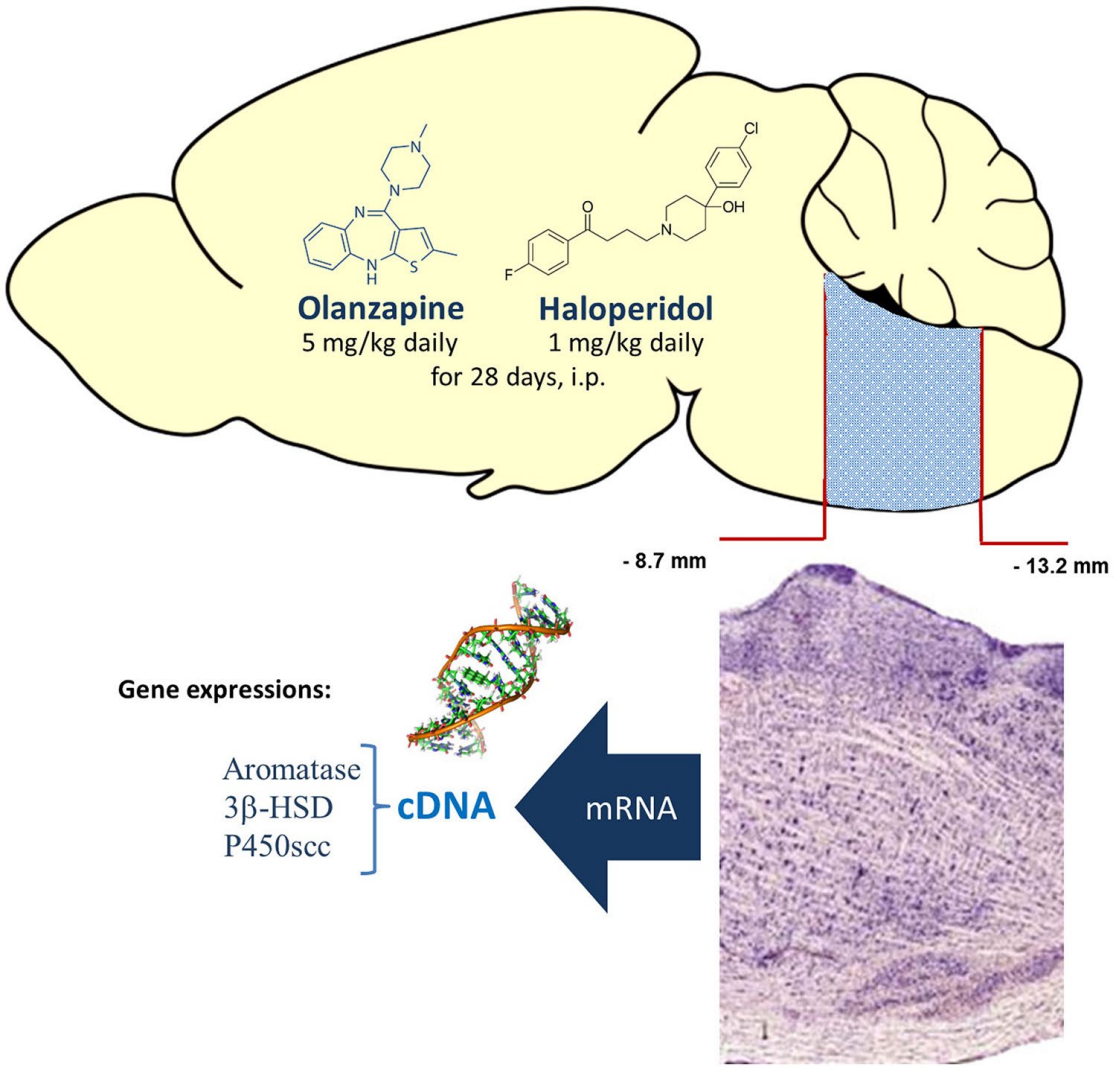
A methodological scheme of the study. The brainstem segments (− 8.7 to − 13.2 mm from bregma) were excised, total mRNA was isolated, transcribed into cDNA and the Real Time-PCR method was used for estimation of related aromatase, 3β-HSD and P450scc gene expressions. Tissue samples contained reticular formation neurons including aminergic and peptidergic cells. Structural figure based on modified sagittal brain sections taken from the standard Paxinos and Watson The Rat Brain Atlas

Adult (5 month old, 210–240 g) male Sprague–Dawley rats (Medical University of Silesia Experimental Centre) were housed at 22 °C with a regular 12/12 light-darkness cycle and with access to standard Murigran chow and water ad libitum. All experimental procedures were approved by the Local Bioethical Committee at the Medical University of Silesia (agreement no. 36/2012) and were conducted in a manner consistent with NIH Guidelines for Care and Use of Laboratory Animals. Individuals were divided into three groups (n = 5)—received olanzapine (5 mg/kg/day) or haloperidol (1 mg/kg/day) or control saline vehicle by intraperitoneal injection for 28 days. 24 h after the last drug administration, rats were anaesthetized using isoflurane and killed. Total mRNA was extracted from excised brainstem sections (each sample was taken from one individual, segments from − 8.7 to − 13.2 mm from bregma, Fig. 2) via the phenol–chloro-form method using Trizol™. Collected mRNA samples were transcribed into cDNA during incubation in a buffered solution of reverse transcriptase MMLV-RT with RNAsin, oligo-dT, and a mix of nucleotides. The whole process occurring at 42 °C for 60 min using DNA Thermal Cycler 480 (Perkin Elmer Inc., Waltham, MA). The quantitative real-time PCR reaction (qPCR) was performed by FastStart SYBR Green Master mix (Roche) in a Light Cycler 1 96 (Roche) with previously prepared cDNA. Glyceraldehyde-3-phosphate dehydrogenase (GAPDH) was chosen as a standard internal reference gene. Transcripts were; amplified using the following primers (Sigma, Life Science): CYP19A1:F:5′–TAAAAGATGGCACACAAAGAGTGC, R: 5′–ACCGAGGTTAC CTGGATCTGC; P450scc: F: 5'-AGAAGCTGGGCAACATGGAGTCAG-3′, R:5'-TCTCATCCCAGGCAGCTGCATGGT-3′; 3β-HSD: F: 5'-ACTGGCAAA TTCTCCATAGCC-3′, R: 5'-CTTCCTCCCAGCTGACAAGTGG-3′; GAPDH: F: 5′-GTGAACGGATTTGGCCGTATCG-3′, R: 5′-ATCACGCCACAGCTTTCCAGAGG-3′. Optimal hybridization temperature was established according to a gradient PCR and was 62, 65, 65 °C respectively and 69 °C for GAPDH housekeeping gene. Statistical analysis was performed using Statistica 10 (Systat software). Data were statistically analyzed using one-way ANOVA and HSD Tukey’s tests. Differences were considered statistically significant at p < 0.01.

## Results and discussion

A growing body of recent evidence points to a key role of neurosteroids in the wide spectrum of brain function. The effect of neurosteroids on the release of neurotransmitters has attracted a lot of attention as this process is the first important step in ensuring effective synaptic transmission [1]. Distribution of steroidogenic enzyme expression is strictly region and cell specific and may possibly determine the local mode of antipsychotic action in the brain.

**Fig. 3:**
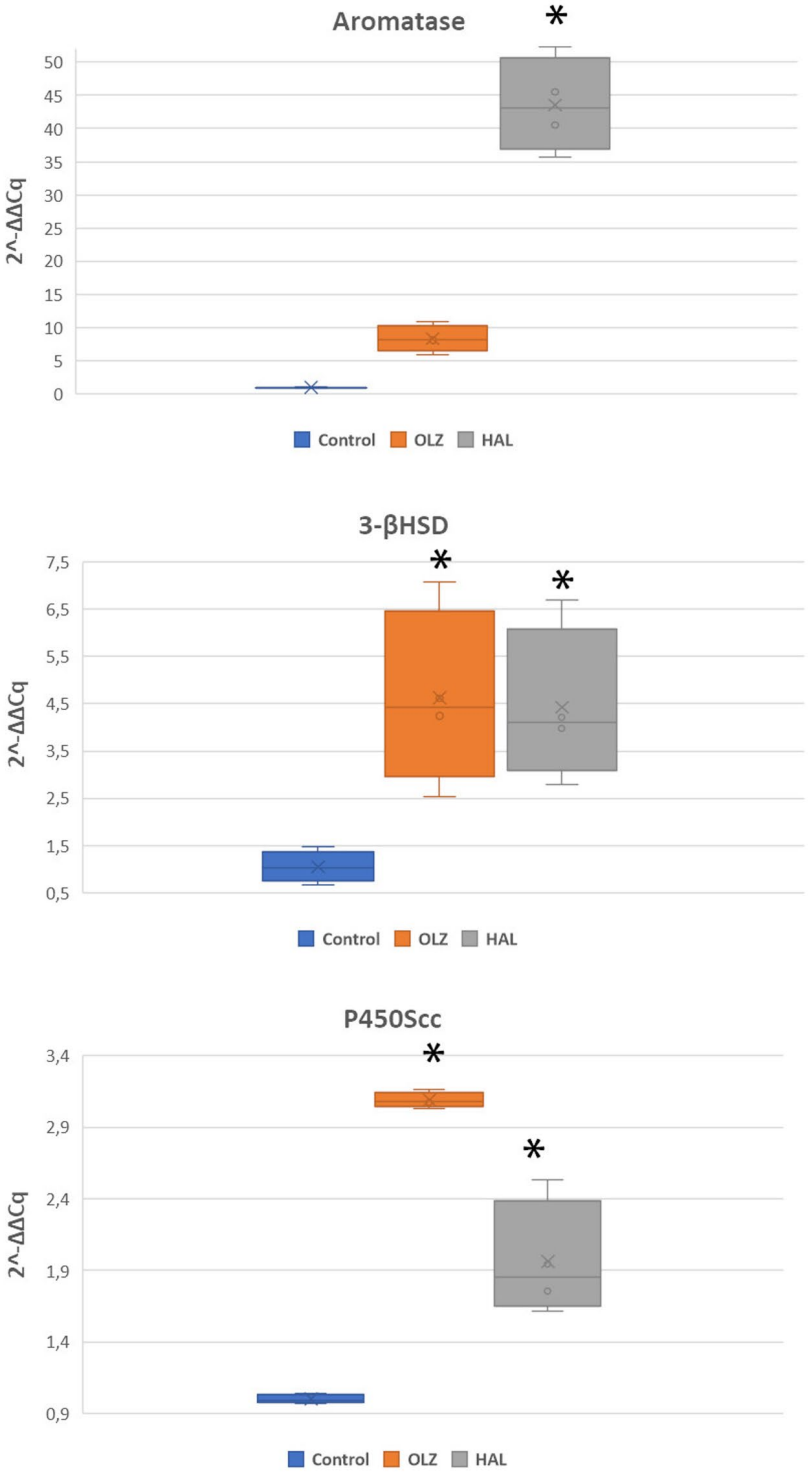
Quantitative real-time PCR results of relative aromatase, 3-βHSD and P450Scc mRNA expression levels in the rat brainstem. Obtained results were normalized to GAPDH housekeeping reference gene. Data are presented as mean 2^−ΔΔCq^. One-way ANOVA followed by Tukey’s HSD post-hoc test was used for statistical analysis (experimental group vs control). p ≤ 0.01 is considered as statistically significant

Our experiment showed for the first time that selected first- and second generation antipsychotics can affect the gene expression of the main neurosteroidogenic enzymes in the rat brainstem. Presented Real-Time PCR data show that after long-term treatment with olanzapine P450scc and 3β-HSD mRNA expression in the rat brainstem were significantly increased (3.09 ± 0.07 vs. control p = 0.00018 and 4.62 ± 2.30 vs. control p = 0.0009 respectively) (Fig. 3). Olanzapine increased the mean of the aromatase mRNA level, more than other enzymes (8.36 ± 2.49) but this effect was not statistically significant (p = 0.081) (Fig. 3). A particular interesting gene activation was found in the brainstem tissue after treatment with haloperidol, with mRNA expression increasing for each type of enzyme but to a different degree. For P450scc we observed lower gene expression than after treatment with olanzapine (1.96 ± 0.4 vs. control p = 0.00018) (Fig. 3), while expression for 3β-HSD mRNA increased to the same level as after treatment with olanzapine (4.43 ± 1.64 vs. control p = 0.0013) (Fig. 3). Most notably, haloperidol increased aromatase mRNA, and it was significantly higher than in the olanzapine treated group (43.53 ± 7.10) vs. control p = 0.000183 (Fig. 3). Although comparable studies on the brainstem steroidogenesis after pharmacomodulation are not available, the presented results are generally in line with our previous studies showing increased level of aromatase expression in the whole brain hemispheres of rats chronically treated with atypical (clozapine) and typical (haloperidol) antypsychotics [9]. They are also partly consistent with other studies reporting a distinct elevation of the cortical and striatal allopregnanolone, allotetrahydrodeoxycorticosterone (THDOC) and 3α,5α-tetrahydroprogesterone (3α,5α-THP) concentrations in rats after short-term clozapine and olanzapine administration [10]. An interesting study reports that both ganaxolone and 3α, 5αα -THP restored citalopram-induced reduction of the dorsal raphe neuron firing activity in the rat brainstem suggesting that neurosteroids can be considered as potential adjuvants in the pharmacotherapy of mood disorders, particularly in female patients [11]. On the other hand, a stimulation of tritium-labeled flunitrazepam binding by 3α-OH-DHP in the brainstem nuclei regulated the baroreflex circuits suggesting that neurosteroids may be involved in blood pressure control [12].

The molecular mechanism of antipsychotics effects on the brainstem steroidogenesis is so far unknown. Possibly, an elevation in aromatase, 3β-HSD and P450scc gene expression may be caused by inhibition of specific dopamine and serotonin receptors located in certain aminergic neurons. Interestingly, an inhibition of brain steroid synthesis with selective blockers of 3β-HSD (trilostane) and 3-a-hydroxysteroid oxidoreductase (indomethacin) and silencing of GABAA receptor transmission with bicuculline abolished the behavioral effects of olanzapine (but not haloperidol and risperidone) administration in rats. This finding does suggest that neurosteroid-dependent modulation of GABA-ergic signalling may play an important role in the neuroleptic activity of olanzapine [13]. A valuable hypothesis by Maria Luisa Barbaccia [14] predicts that antipsychotics, as well as some antidepressants, increase neurosteroid levels in several brain structures which supports the aforementioned considerations.

**Fig. 4:**
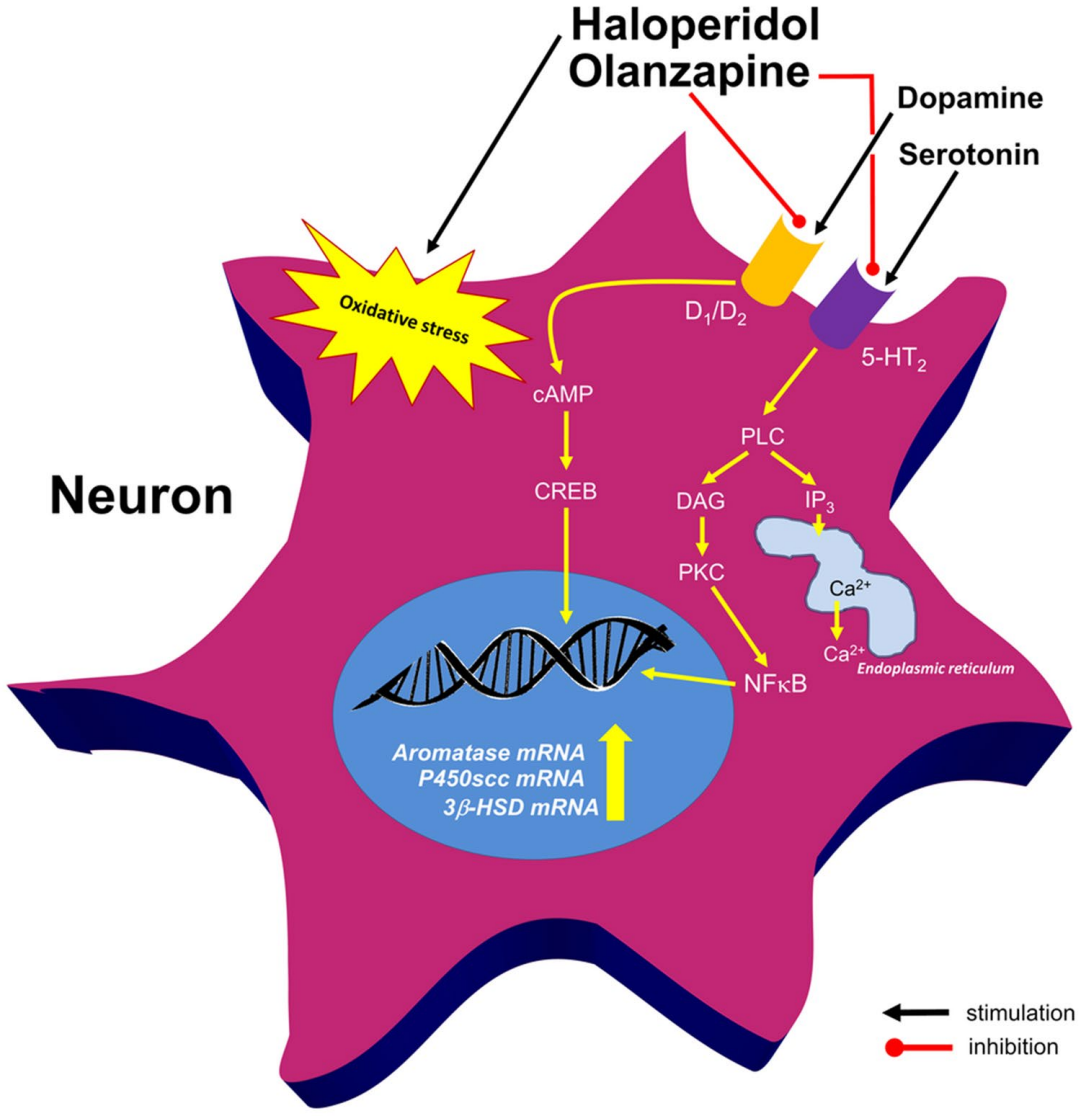
A possible mechanism of the effect of olanzapine and haloperidol on aromatase, 3β-HSD and P450scc expressions in the rat brainstem. The steroidogenic enzyme level in some neurons can probably be modulated by their dopamine and serotonin, receptors. Haloperidol-related oxidative stress may in turn stimulate neuronal aromatase expression that can possibly be a kind of protective response against antipsychotic toxicity. Olanzapine may increase the gene expressions of steroidogenic enzymes through the inhibition of both dopamine D_1_/D_2_ and serotonin 5-HT_2_ receptors. This may block cAMP and PLC- dependent signalling and modulate gene transcription. *cAMP* cyclic AMP, *CREB* cAMP response element binding protein, *DAG* diacylglycerol, *IP*_*3*_ inositol triphosphate, *NFkB* nuclear factor kappa-light-chain-enhancer of activated B cells, *PKC* protein kinase C, *PLC* phospholipase C

Several other possibilities can be taken into consideration to explain how antipsychotics modulate steroidogenic enzymes synthesis. Hypothetically, an elevation in enzyme mRNA expressions may be caused by blockade of selective monoamine, mainly dopamine receptors located in certain neuronal and glial populations (Fig. 4). Nevertheless, selective D_1_ agonist flupentixol caused c-AMP-dependent upregulation of aromatase mRNA expression on cultured goldfish radial glial cells [15]. Moreover, in vitro studies showed that brain aromatase activity was inhibited by both D_1_/D_2_ agonists (apomorphine) and antagonists (spiperone, primozide), suggesting that the observed changes are independent from dopamine receptor action [16]. Noteworthy, sulpiride, a selective D_2_ antagonist affects neurosteroid-induced modulation of evoked [^3^H]-noradrenaline release [17]. On the other hand, extended treatment with fluoxetine, a selective serotonin reuptake inhibitor increased allopregnanolone level in the rat prefrontal cortex and hippocampus, including in animal models of depression [18]. Because the presence of glutamate receptors in the aromatase-expressing cells has been found, a glutamatergic hypothesis of olanzapine action may also be suggested. Nevertheless, pharmacological stimulation with glutamate agonists resulted in reduction of aromatase activity in rodent hypothalamus [19], which is conflicting to the mechanism proposed above. There are also reports that hippocampal aromatase activity and local synthesis of estradiol or pregnenolone may be stimulated by NMDA-dependent calcium influx into the neuroplasm [20]. On the other hand, endogenous pregnenolone facilitated glutamate release in the rat hippocampus [21]. Neurosteroids may therefore act as paracrine modulators of neural transmission within several brain structures not excluding the brainstem. Interestingly, olanzapine is considered to promote the initial phase of neurosteroid synthesis by the stimulation of P450scc mRNA expression. Haloperidol, seems in turn to highly enhance the aromatase mRNA expression. Both two dopamine antagonists seem to act on different regulatory pathways but commonly increase the gene expression levels of examined steroidogenic enzymes in the rat brainstem. The molecular mechanism of these effects is so far unknown. Possibly P450scc synthesis may be regulated via olanzapine binding to several serotonin receptors rather than dopaminergic blockade.

Neurosteroidogenic enzymes may play an important role in the regulation of possible neuroprotective activity of estrogens e.g. an elevation of aromatase activity was reported in the rat cortical structures and basal ganglia after several brain injuries [22]. In the present study extended treatment with haloperidol and olanzapine increased aromatase, 3-βHSD and P450scc mRNA expression in the rat brainstem. It should be pointed out that haloperidol as well as other first generation antipsychotics exhibit several neurotoxic actions e.g. it can dysregulate neuronal oxidative balance [6] Moreover, a longitudinal haloperidol administration disturbed glutamatergic transmission in the rat prefrontal cortex, that was mainly an effect of NMDAR activity inhibition caused by decreased GluN1 and GluN2A but not GluN2B subunit expression [23]. Possibly, the elevation of aromatase mRNA expression observed in our study may be part of a neuronal protective mechanism against an extended treatment with this drug.

On the other hand, olanzapine has a high affinity to several central, peripheral and even non neuronal types of dopamine and serotonin receptors [6].Given that it was administered intraperitoneally, an alteration of gastrointestinal serotonin-secreting cells (ECL) activity can potentially occur that could indirectly influence our results. Moreover, antipsychotic-related changes in steroidogenic enzyme expression found in the brainstem may also result from a compensatory mechanism for a different effect in another endocrine organ e.g. adrenal glands and testes, as steroids can easily pass through the blood–brain barrier. For instance, olanzapine and to a lesser extent aripiprazole administration may directly decrease FSH, LH and testosterone levels and disturb spermatogenesis in male rats [24]. This effect may potentially be related with an inhibition of 3-βHSD activity in the Leydig cells.

It should be pointed out that there are some limitations to our study. For instance enzyme protein levels in the brainstem nuclei were not measured and the estimation of estrogen levels were not provided. However, these preliminary novel findings should be treated as an initial step in the pharmacological interplay between antipsychotic action and neurosteroidogenesis at the level of brainstem centres.
